# Supplementary figures and images for: Infant Botulism: Checklist for Timely Clinical Diagnosis and New Possible Risk Factors Originated from a Case Report and Literature Review
Source: Toxins (Basel). 2021 Dec 2;13(12):860. doi: 10.3390/toxins13120860 (PMC8703831; doi:10.3390/toxins13120860)

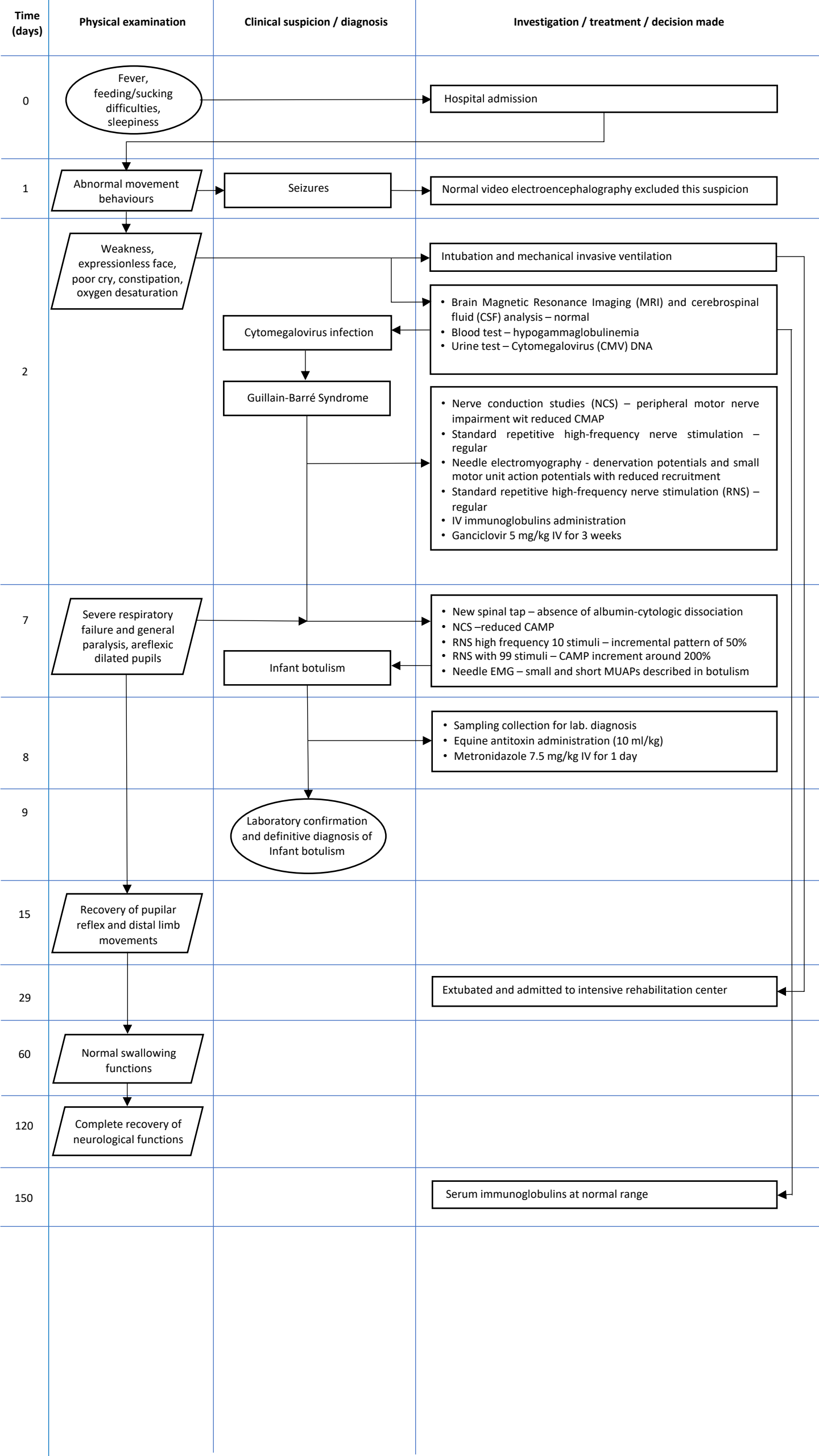

Supplement: Supplementary file 1 [file toxins-13-00860-s001.zip › toxins-1482327-supplementary.pdf]
